# Primary Cough Headache: A Case Report

**DOI:** 10.7759/cureus.36971

**Published:** 2023-03-31

**Authors:** Khudheeja A Ahmed, Juwayria A Ahmed, Ibrahim Mohsin, M. Habeeb Ahmed

**Affiliations:** 1 Research, KAAJ Healthcare, San Jose, USA; 2 Internal Medicine, Norton Community Hospital, Norton, USA; 3 Cardiology, KAAJ Healthcare, San Jose, USA

**Keywords:** case report, evolution of symptoms, indomethacin, valsalva maneuver, primary cough headache

## Abstract

Primary cough headache (PCH) is an uncommon condition characterized by episodes of sudden onset, bilateral headaches typically lasting from a second to two hours. Headaches are notably associated with Valsalva maneuvers such as coughing or straining but not a prolonged physical exercise in the absence of intracranial abnormalities. We report an unusual presentation of PCH in a 53-year-old female suffering from episodes of severe sudden-onset headaches that lasted several hours. The headaches were initially precipitated by coughing as is typical with PCH but were unusual in the way the triggers for the episodes evolved. Headaches began occurring without any association of Valsalva maneuvers and ultimately occurred with no discernible trigger. The patient initially came to the cardiologist's office who then referred her to a neurologist for further evaluation. The neurologist initially prescribed methylprednisolone tablets, primarily to suppress the cough. Magnetic resonance imaging, magnetic resonance angiography (MRA) of the brain, and a head CT scan were then performed to rule out possible secondary causes such as a mass, intracranial bleed, aneurysms, or other vascular anomalies. The neurologist prescribed indomethacin and topiramate four and nine days after diagnosis of PCH, respectively. After five days, the beta blocker metoprolol tartrate was prescribed as the patient's blood pressure was rising significantly in correlation to the headaches. The above treatment was effective in limiting the intensity and duration of the headaches and the symptoms resolved after four weeks. This case contributes towards the understanding of the potential evolution of PCH presenting with triggers unassociated with Valsalva maneuvers and ultimately occurring with no known trigger as well as providing an example of PCH with an unusually long duration.

## Introduction

Cough headaches are an uncommon type of head pain triggered by coughing or straining. Cough headaches are subdivided into primary and secondary cough headaches [1]. Secondary cough headache (SCH) may be triggered by coughing but is caused by an underlying condition [2]. Approximately 40% of cases are found to have underlying pathologies, most commonly Chiari type 1 malformation, as well as posterior fossa lesions, cerebral aneurysms, obstructive hydrocephalus, and spontaneous low cerebrospinal fluid (CSF) pressure [2]. A neuroimaging evaluation, preferably a brain MRI with and without contrast is necessary for patients presenting with a cough headache [3]. Once all underlying etiologies are ruled out, only then may a cough headache be considered a primary cough headache (PCH) [1]. The diagnostic criteria for PCH, also known as Valsalva maneuver headache, are described by the International Headache Society (IHS) as: at least two sudden onset headache episodes lasting between one second and two hours; brought on “only in association with coughing, straining, and/or other Valsalva maneuver” in the absence of any intracranial disorder [1]. Primary cough headache is bilateral in distribution (but can be unilateral) and pain ranges from moderate to severe intensity and is described as being sharp and stabbing in quality [1-3]. The headache reaches peak intensity almost immediately and typically “subsides over several seconds to a few minutes” [1]. A minority of patients have reported headaches lasting up to two hours [4]. Vertigo, nausea, and sleep abnormalities were found to be associated symptoms for up to two-thirds of patients with PCH but vomiting, light or sound sensitivity, and lacrimation are not generally associated with PCH [1,3]. Primary cough headache is most often found in people over 40 years of age, and the average age at presentation is about 60 years of age [3]. Though it was initially thought to be more common in men than in women, Pascual et al. found in a 10-year study that sex is not correlated to being affected by PCH [5]. 

Primary cough headache symptoms can be debilitating and a preventative treatment strategy should be considered [2]. First, any pulmonary disease causing chronic coughing must be addressed [3]. Indomethacin is the drug of choice for PCH [1-3]. Several studies have found 25 mg to 150 mg daily doses for about two to five months to be an effective treatment and another study found that up to 250 mg daily may be required [2]. The possible mechanism of action for indomethacin may be that it reduces intracranial pressure [2,3]. Both acetazolamide and cerebrospinal fluid drainage reduce intracranial pressure and have been effective at bringing about a significant improvement or complete remission of PCH symptoms within a majority of patients undergoing these treatments [2,3]. Other treatment options have been reported to be beneficial in some smaller case series, including topiramate, propranolol, methysergide, naproxen, ergonovine, intravenous dihydroergotamine, and phenelzine [2,3]. We document an unusual case of PCH presenting with triggers unassociated with Valsalva maneuvers and episodes occurring with no evident trigger over time, providing insight into the variety and possible presentations of PCH. 

## Case presentation

A 53-year-old female presented to the cardiologist’s clinic with a splitting headache precipitated by Valsalva maneuvers. The headache was bilateral in distribution, high in intensity, and characterized by splitting or stabbing pain. The patient described the pain as significantly more severe than labor pain and stated that it was the worst headache of her life. Symptoms included the sensation of an electric current running down the arms and hands. The patient does not take any chronic medication nor has any past medical history of either hypertension, smoking, or drug or alcohol use. The patient has a lifelong history of very occasional low-grade headaches occurring intermittently over the years, but never as severe or debilitating as the current attacks. She is a teacher by profession. 

The attacks had started several days prior and were first triggered by coughing, defecation, and urination, all types of Valsalva maneuvers. The duration of attacks was typically about 50 minutes, and a duller, more moderate headache lasted up to three to five hours afterward. The frequency of attacks was three to four times daily, collectively occurring throughout most of the day. Attacks were not triggered by walking. Blood pressure was recorded, which was rising to 185/105 mmHg usually about 20 minutes after an attack. Two hours after the attack, blood pressure decreased to about 150/85 mmHg, and then decreased to an average of 140/80 mmHg over the next several hours. The pulse rate during the attacks was around 70 bpm and decreased to about 60 bpm four to five hours after the attack. The patient took acetaminophen 500 mg as needed during previous attacks. 

**Figure 1:**
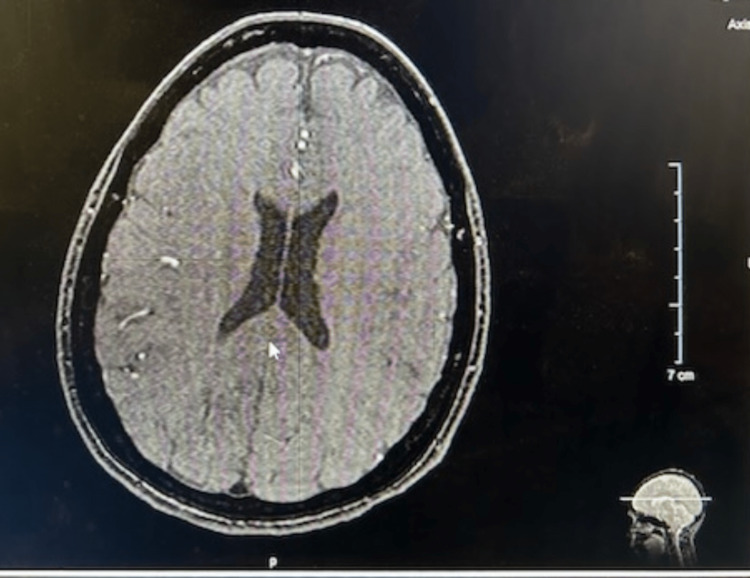
The MRI of the brain (axial view) showed no relevant or significant changes

**Figure 2:**
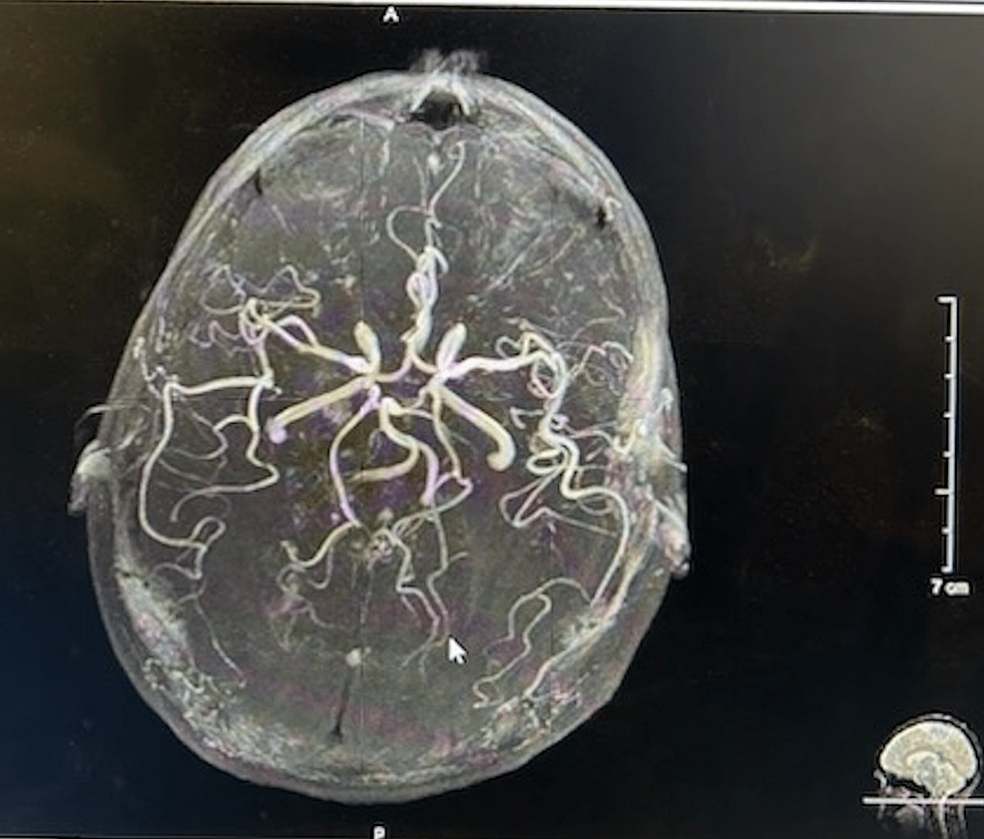
The MRA of the head described no hemodynamically significant stenosis or aneurysm. No vascular malformation were observed in the intracranial circulation, nor were there other relief changes. MRA: Magnetic resonance angiography

**Figure 3:**
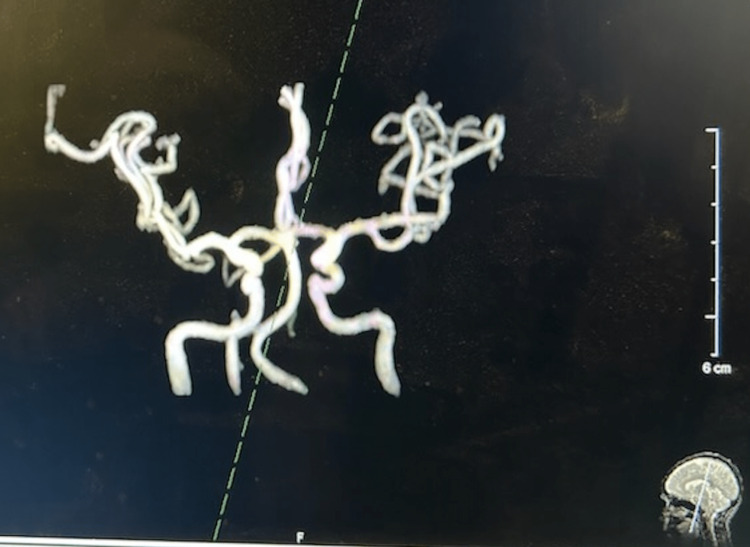
A closer look at the MRA of the head shows no hemodynamically significant stenosis, aneurysm, vascular malformation in the intracranial circulation, or other relief changes. MRA: Magnetic resonance angiography

**Figure 4:**
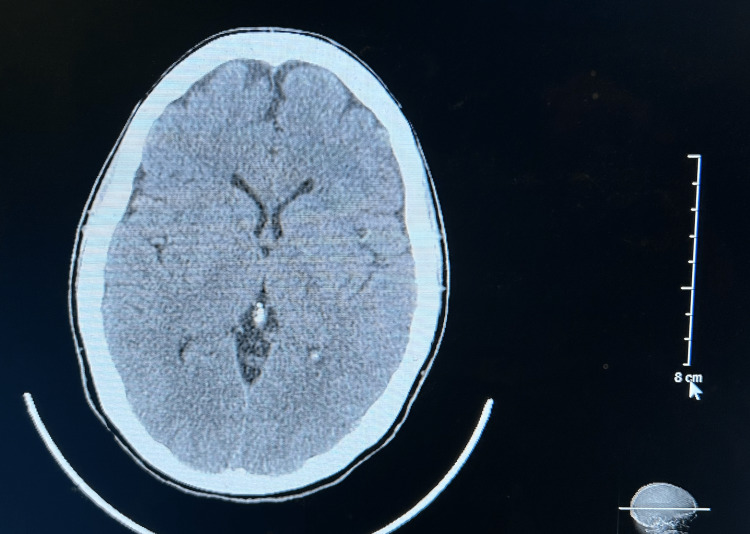
The CT scan of the brain (axial view) showed no relevant or significant changes

Because of the acute onset of headaches and patient complaints, the patient was referred to a neurologist and examined. She denied any blurring of vision, facial numbness, neck pain, back pain, tingling or numbness of the arms, dizziness, and loss of balance. In the general and neurological exams, no changes were identified. There was no neck rigidity or any focal deficits. To suppress the cough, the neurologist prescribed a six-day sequence of 4 mg methylprednisolone tablets, starting at six a day for the first day, then five a day for the second day, dropping continuously until reaching one a day by the sixth day. Diagnostic neuroimaging was performed to assess for structural abnormalities and possible secondary etiologies. A brain MRI (Figure 1) with and without contrast, magnetic resonance angiography (MRA) of the brain (Figures 2, 3), and a head CT (Figure 4), were performed. All scans came back negative for structural lesions or abnormalities. There was no evidence of Chiari type-1 malformations, tumors, or cerebral aneurysms. 

 

Bloodwork was within normal parameters. Laboratory investigations showed white blood cell count at 6.7 thousand/uL, erythrocyte count of 4.86 million/uL, hemoglobin 13.3 g/dL, hematocrit 40.4%, platelet count at 365 thousand/uL, thyroid-stimulating hormone (TSH) level at 1.60 mIU/L, glucose level of 105 mg/dL, sodium 139 mmol/L, potassium 3.8 mmol/L, chloride 106 mmol/L, calcium 9.0 mg/dL, blood urea nitrogen (BUN) 16 mg/dL, and creatinine at 0.59 mg/dL. 

After the underlying pathologies of a SCH were ruled out through diagnostic neuroimaging, the patient was diagnosed to have a PCH. Four days after the diagnosis, headache triggers began to change. The patient was prescribed butalbital, acetaminophen, and caffeine combination capsules of 50 mg, 325 mg, and 40 mg, respectively, as needed and indomethacin 50 mg thrice daily. Instances of chewing and contact with cold surfaces also triggered the headaches. Five days after diagnosis, the patient was prescribed topiramate 25 mg twice daily due to recurring headaches. The patient was also prescribed metoprolol 25 mg daily which was increased to 50 mg to address increased blood pressure levels. Ten days after the initial diagnosis, the headaches were precipitated by no apparent trigger.

After about two weeks of treatment, the duration and frequency of the headache began to decrease. Initial episodes of about 50 minutes of severe and splitting pain were limited to about five to 10 minutes, and the frequency of episodes was about twice daily. A more moderate headache lingered after each attack, but the pain was reduced in intensity compared to the aftereffects of earlier attacks. Since blood pressure levels still neared 190/100 mmHg during the attacks and remained as high as 160/90 mmHg several hours after the episodes, the metoprolol dosage was increased to 75 mg daily. 

Since the attacks were decreasing in frequency, the indomethacin dosage was tapered off over four days, and the topiramate dosage was doubled. The patient reported side effects of disorientation and confusion, and therefore topiramate dosage was reduced to 25 mg daily. By the third week after the diagnosis, the splitting headache attacks had ceased to occur. Blood pressure levels were recorded at 140/80 mmHg with a pulse of 60 bpm. By the end of the third week, the patient's headaches reduced in intensity from severe to a persistent moderate headache lasting throughout the day. Blood pressure levels returned to normal levels of below 120/80 mmHg and were being maintained by metoprolol 50 mg twice daily for one month. However, over two months, hypertension had resolved with blood pressure readings running at 90 mmHg to 110 mmHg systolic, and metoprolol was no longer prescribed. 

## Discussion

The etiology of PCH is not clearly understood. Cordenier et al. postulate that coughing causes sudden increases in intra-abdominal and intra-thoracic pressures subsequently leading to an increase in the central venous pressure and the activation of nociceptive neurons [2]. Pascual et al. theorize that other contributing factors may include hypersensitivity of pressure-sensitive receptors near venous vessels, the cause of which could be a hidden or a previous infection [5]. Chen et al. discovered that patients with PCH are associated with a more crowded posterior cranial fossa [4]. 

This case report adds more insight into the presentation, evolution, and treatment of PCH. The definition of PCH under the IHS states that headache attacks typically do not last past two hours [1]. Primary cough headaches usually range from several seconds to several minutes [1]. A comprehensive 10-year study found that PCH pain typically only lasts seconds [5]. This case report shows a patient whose duration of headache attacks lasted for about one to three hours. The patient also had duller, more moderate headaches lasting up to three to five hours after each attack. There has been some documentation of cases with atypical headache durations. Chen et al. published a series of 74 PCH patients where the median headache duration was thirty seconds but was greater than thirty minutes and up to two hours in about 11% of patients [4]. Our patient’s unusually extended duration of headache attacks shows that the presentation of PCH may differ and that headache duration may be inconsistent and longer than the IHS criteria. 

Most noteworthy for this discussion, we found that the triggers precipitating the headache in this patient changed over time, and new triggers developed. Initially, the headaches were triggered by coughing, straining, and other Valsalva maneuvers. However, symptoms changed gradually to also include contact with cold floors and minor actions such as chewing. Even more unusually, the patient ultimately experienced headaches that appeared to have no obvious trigger. This evolution of symptoms is highly unusual in the case of PCH. The definition of PCH states only that Valsalva maneuvers trigger headaches, and evolution of triggers is not included [1]. 

In the literature, we have found no known cases of this type of evolution in the triggers of PCH. There are reported cases of patients whose nature of symptoms evolved, such as a change in location within the head, or change in severity but in all cases, the headaches remained associated with coughing, straining, stooping, and/or other Valsalva maneuvers [6]. Pascual et al. found in a comprehensive 10-year study that 100% of PCH patients experienced pain provoked by coughing, with 56% by sudden postural movements, and 22% by defecating, but in all cases researchers did not observe pain occurring without provocation by Valsalva maneuvers [5]. We found one similar case of a 65-year-old male who presented with severe sudden onset headaches of a shooting or throbbing nature with no observed abnormal signs other than similarly elevated blood pressure readings of 205/105 mmHg. He was given a nasal douche to treat catarrh and prevent excessive coughing after which his headaches resolved within a few months [6]. 

Our case report is particularly beneficial because it describes an example of a PCH patient’s evolution of symptoms, providing documentation of PCH triggers shifting from Valsalva maneuvers to variable causes and ultimately having no discernible trigger. Our patient's treatment began with 50 mg of indomethacin, thrice daily. As recurring headaches persisted, topiramate (50 mg daily) was prescribed as well. Indomethacin was prescribed for two weeks after the diagnosis and then tapered off. Topiramate was prescribed for three weeks after diagnosis and ended when the headache attacks resolved. Under this broad treatment, we observed a reduction in headache frequency, duration, and intensity of pain. The treatment of choice in the literature is indomethacin. Several studies of varying treatment durations found that daily doses ranging from 25 mg to 150 mg were effective [2]. There is no consensus on treatment duration in the literature [2]. Pascual et al. found that a maximum of five months was required to resolve the headache attacks [5]. Chen et al. found that every patient was pain-free six months after indomethacin was started [4]. Topiramate has also been found to be of benefit [7]. We found that the headache attacks decreased most in frequency and intensity after topiramate was started. We also found metoprolol (100 mg daily) to be effective as a short-term measure in addressing the elevated blood pressures that were correlated with the incidence of headache attacks. 

Although a limited number of longterm studies exist on the natural evolution of cough headaches, Symonds reports that all 21 observed cases spontaneously recovered within a few months to five years, with one exception of 12 years [6]. Pascual et al. found that the mean duration of the symptomatic phase was 11 months, with a range of one to 42 months [5]. One Danish study has estimated the lifetime prevalence of PCH to be at 1% in a survey of a representative 25 to 64 years old general population [8]. In our case, PCH resolved within four weeks, with headaches almost disappearing by the third week after the diagnosis. Therefore, this duration is consistent with the literature.

## Conclusions

Primary cough headache is defined by sudden onset, splitting headache precipitated by Valsalva maneuvers in the absence of any underlying etiologies. We report a unique case of a 53-year-old female diagnosed with PCH with symptoms of sudden onset severe headaches occurring with evolving triggers and eventually occurring without association to any Valsalva maneuvers, which is inconsistent with the IHS description. Headaches lasted up to several hours, and the patient exhibited an unusual evolution of symptoms since the headache triggers changed over time until the patient ultimately experienced headache attacks with no known trigger. Treatment with indomethacin and topiramate lasted for four weeks and was found to be beneficial. The patient's PCH symptoms of severe, sudden onset headaches resolved within four weeks, after which therapy ended. This case report adds insight into the variety of presentations of PCH and the possible evolution of triggers as well as the documentation of the unusually long duration of headache episodes. We hope that the information presented in this case report can add to the understanding of how PCH can present, evolve, and respond to different treatment medications, and help guide other clinicians in their diagnostic and therapeutic process.

## References

[1] Headache Classification Committee of the International Headache Society (IHS) (018). The International Classification of Headache Disorders, 3rd edition. Cephalalgia.

[2] Cordenier A, De Hertogh W, De Keyser J, Versijpt J (2013). Headache associated with cough: a review. J Headache Pain.

[3] Cutrer FM (2023). Primary cough headache. UpToDate.

[4] Chen PK, Fuh JL, Wang SJ (2009). Cough headache: a study of 83 consecutive patients. Cephalalgia.

[5] Pascual J, González-Mandly A, Martín R, Oterino A (2008). Headaches precipitated by cough, prolonged exercise or sexual activity: a prospective etiological and clinical study. J Headache Pain.

[6] Symonds C (1956). Cough headache. Brain.

[7] Medrano V, Mallada J, Sempere AP, Fernández S, Piqueras L (2005). Primary cough headache responsive to topiramate. Cephalalgia.

[8] Rasmussen BK, Olesen J (1992). Symptomatic and nonsymptomatic headaches in a general population. Neurology.

